# Supplementary material for: MetaRibo-Seq measures translation in microbiomes
Source: Nat Commun. 2020 Jun 29;11:3268. doi: 10.1038/s41467-020-17081-z (PMC7324362; doi:10.1038/s41467-020-17081-z)
Supplement: Supplementary file 10 — Supplementary Data 7 [file 41467_2020_17081_MOESM10_ESM.zip › File2/Confidence_High_Taxonomy/118798_out.krona.html]

Javascript must be enabled to view this page.

members
magnitude
magnitudeUnassigned
count
unassigned
taxon
rank

118798\_out

49

superkingdom
49
2

46
1239
phylum


SRS143895\_contig\_number\_38979
species
1263000
1


SRS049995\_contig\_number\_16278
species
1263004
1

186801
44
class

order
186802
44

family
43
541000

species

SRS011134\_contig\_number\_contig-100\_1162.283438SRS012849\_contig\_number\_20014SRS013951\_contig\_number\_contig-100\_1459.186011SRS015663\_contig\_number\_27070SRS016203\_contig\_number\_9491SRS017821\_contig\_number\_contig-100\_997.173761SRS017916\_contig\_number\_6145SRS019161\_contig\_number\_30211SRS020394\_contig\_number\_contig-100\_331.114795SRS022609\_contig\_number\_19854SRS024132\_contig\_number\_contig-100\_3717.237170SRS045004\_contig\_number\_22650SRS045645\_contig\_number\_6837SRS047433\_contig\_number\_21799SRS048164\_contig\_number\_contig-100\_742.182212SRS049446\_contig\_number\_contig-100\_1757.94795SRS049959\_contig\_number\_18278SRS051031\_contig\_number\_27416SRS052697\_contig\_number\_14158SRS058070\_contig\_number\_2100SRS063518\_contig\_number\_28326SRS064757\_contig\_number\_2084SRS075341\_contig\_number\_23787SRS075773\_contig\_number\_13819SRS078176\_contig\_number\_11253SRS101376\_contig\_number\_3734SRS1041092\_contig\_number\_3167SRS1041133\_contig\_number\_21872SRS1041136\_contig\_number\_12490SRS1041140\_contig\_number\_7981SRS1041141\_contig\_number\_13486SRS1041145\_contig\_number\_23962SRS104400\_contig\_number\_11696SRS104975\_contig\_number\_contig-100\_4610.100970SRS1055083\_contig\_number\_12006SRS142503\_contig\_number\_43595SRS144537\_contig\_number\_6826SRS147271\_contig\_number\_35784SRS147346\_contig\_number\_contig-100\_2284.319199SRS147557\_contig\_number\_13136SRS149784\_contig\_number\_17799SRS150029\_contig\_number\_12264
42
1898205

216851
1
genus

1262897
1

SRS018836\_contig\_number\_21654
species

family
1
31979

genus
1
1485

1650661
1
species

SRS015782\_contig\_number\_25736

976
3
phylum

class
3
200643

3
171549
order

family
1
1853231

574697
1
genus

1
1969738

SRS971276\_contig\_number\_6027
species

815
2
family


SRS1041032\_contig\_number\_contig-100\_3301.3302
genus
816
2
1

357276
1

SRS018541\_contig\_number\_6186
species
